# Role of Serum Vascular Endothelial Growth Factor (VEGF) as a Potential Biomarker of Response to Immune Checkpoint Inhibitor Therapy in Advanced Melanoma: Results of a Pilot Study

**DOI:** 10.3389/fonc.2020.01041

**Published:** 2020-06-30

**Authors:** Muhammad A. Khattak, Afaf Abed, Anna L. Reid, Ashleigh C. McEvoy, Michael Millward, Melanie Ziman, Elin S. Gray

**Affiliations:** ^1^Department of Medical Oncology, Fiona Stanley Hospital, Murdoch, WA, Australia; ^2^School of Medical and Health Sciences, Edith Cowan University, Perth, WA, Australia; ^3^School of Medicine, University of Western Australia, Crawley, WA, Australia; ^4^Department of Medical Oncology, Sir Charles Gairdner Hospital, Nedlands, WA, Australia

**Keywords:** immunotherapy, vascular endothelial growth factor, melanoma, biomarker, ipilimumab, pembrolizumab, nivolumab

## Abstract

**Background:** The development of biomarkers predictive of response to immune checkpoint inhibitor (ICI) therapies in advanced melanoma is an area of great interest in oncology. Our study evaluated the potential role of serum vascular endothelial growth factor (VEGF) as a predictive biomarker of clinical benefit and response to treatment with ICIs.

**Methods:** Pre-treatment peripheral blood samples were obtained from advanced melanoma patients undergoing ICI therapy as monotherapy or in combination at two tertiary care hospitals in Western Australia. Serum VEGF levels were correlated with response to therapy and survival outcomes.

**Results:** Serum VEGF samples were collected from a total of 130 patients treated with ICI therapy (pembrolizumab 73, ipilimumab 15, and ipilimumab/nivolumab combination 42). Median serum VEGF level was significantly higher in the non-responders (82.15 pg/mL) vs. responders (60.40 pg/mL) in the ipilimumab monotherapy cohort (*P* < 0.0352). However, no difference was seen in VEGF levels between non-responders and responders in pembrolizumab and ipilimumab/nivolumab treated patients.

**Conclusions:** The results of our study confirm previous observations that that high pre-treatment serum VEGF levels in advanced melanoma patients may predict poor response to ipilimumab. However, serum VEGF is not predictive of outcome in patients treated with anti-PD-1 agents alone or in combination with ipilimumab.

## Introduction

Immune checkpoint inhibitors (ICIs) have improved the survival of advanced melanoma patients with a 5 year survival rate of about 50% (1, 2). However, a substantial proportion of patients still do not benefit from this treatment. Biomarkers predictive of response to ICI therapy in advanced melanoma are lacking. This has important implications as ICI therapy is costly and can be associated with immune adverse events leading to serious patient morbidity (3).

A number of tumour and host related biomarkers predictive of response to immunotherapy are currently being evaluated (4). Although tissue-based biomarkers have been traditionally considered the gold standard for predicting response to treatment (5), a number of blood-based biomarkers are being increasingly utilized in immuno-oncology (6). Serum lactate dehydrogenase (LDH), neutrophil lymphocyte ratio, serum albumin, and C reactive protein (CRP) have been evaluated for their prognostic significance (7, 8). Blood based markers are relatively non-invasive with an advantage of longitudinal sample collection allowing us to potentially track an evolving anti-tumour immune response. One such marker is serum vascular endothelial growth factor (VEGF) (9).

Vascular endothelial growth factor (VEGF) mediates immunosuppression through inhibition of dendritic cell maturation, reduction of cytotoxic T-cell infiltration into tumour and by creating an immunosuppressive tumour microenvironment through upregulation of myeloid derived suppressor cells (MDSCs) and regulatory T-cells (Tregs) (10, 11). Combination of ICIs and anti-VEGF therapies has shown synergy and positive outcomes in early clinical trials and this approach is being evaluated in a number of trials across a range of tumour types (10).

Yuan et al. (9) have previously demonstrated that pre-treatment serum VEGF levels were associated with poor clinical response and overall survival in advanced melanoma patients treated with ipilimumab. The aim of our study was to evaluate and further validate the prognostic biomarker significance of serum VEGF levels in advanced melanoma patients treated with anti-CTLA4 (ipilimumab), anti-PD1 agents (pembrolizumab/ or nivolumab) or the combination of ipilimumab and nivolumab.

## Patients and Methods

### Patient Selection

Advanced melanoma patients undergoing ICI therapy as monotherapy (ipilimumab, pembrolizumab or nivolumab) or combination therapy (ipilimumab and nivolumab) were recruited from two hospitals in Perth, Western Australia. Participants signed informed consent in accordance with protocols safeguarding patient rights. All procedures were accepted by the Human Research Ethics Committees at Edith Cowan University (ECU) (No.11543) and Sir Charles Gairdner Hospital (No. 2013-246).

Tumour responses were assessed radiologically by CT and/or PET scans at two to three monthly intervals. Scans were reported by radiologists blinded to patient treatment data. Patients were defined as responders if they had significant reduction in tumour size by RECIST 1.1 on CT or PET FDG avidity as per the treating clinician or stable disease lasting more than 6 months. Progression free survival (PFS) was defined as the time interval between the start of therapy and the date of first progression. Overall survival (OS) was defined as the time interval between the start of therapy and death.

### Measurement of Serum VEGF

Peripheral blood samples were obtained from each patient prior to commencement (baseline) of treatment. Serum samples were collected in SST tubes to assess serum concentrations of VEGF. Samples were used undiluted and in duplicate, using a Milliplex® MAP Human Cytokine/Chemokine Magnetic Bead Panel kit (EMD Millipore, USA) for 96 well plate assay using the protocol provided by the manufacturer. xPONENT® software (Luminex Corp., USA) was used to detect, quantitate, and analyse the samples on the Luminex 100™ instrument.

### Statistical Analysis

Non-parametric Mann-Whitney *U*-tests were used to compare VEGF levels. Patients were dichotomized based on high and low serum VEGF concentrations. A cut-off value was calculated for the ipilimumab treated cohort using CutoffFinder (12). Survival curves were plotted using the Kaplan-Meier method and hazard ratios computed through a Mantel-Cox analysis. Analyses were performed using GraphPad Prism 7 (GraphPad Software, San Diego, CA, USA) and IBM SPSS Statistics version 25 (IBM, Armonk, NY, USA).

## Results

Serum samples were collected from a total of 130 patients treated with ICI therapy as follows: pembrolizumab 73, ipilimumab 15, and ipilimumab/nivolumab combination 42. Baseline patient characteristics are summarized in Table 1.

**Table 1:** Patient characteristics.

	***N***	**%**
**Total**	129^*^	
**Age (yrs)**		
<70	83	64
≥70	46	36
**GENDER**		
M	92	71
F	37	29
**STAGE**		
IIIC/D (unresectable)	9	7
IV (M1a)	17	13
IV (M1b)	15	12
IV (M1c)	57	44
IV (M1d)	31	24
**BRAF STATUS**		
Wild type	82	64
Mutant	46	36
Unknown	1	1

* *data not evaluable for one patient*.

Serum levels VEGF was similar between the patient groups, with 32–450 pg/mL in the ipilimumab group, 25–330 pg/mL in anti-PDL1 treated group, and 24–490 pg/mL in the combination group.

**Figure 1:**
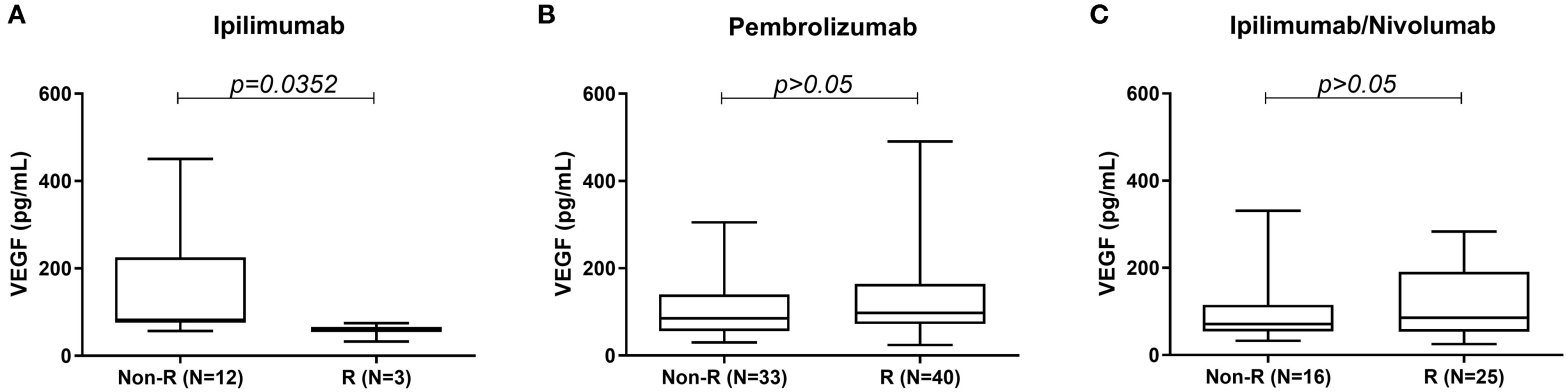
Comparison of serum VEGF levels between responders and non-responders to **(A)** ipilimumab, **(B)** pembrolizumab, and **(C)** ipilimumab/nivolumab treatment.

High serum VEGF level was associated with poor response to treatment in the ipilimumab cohort. Median serum VEGF level was significantly higher in the non-responders (82.15 pg/mL) vs. responders (60.40 pg/mL) in the ipilimumab monotherapy cohort (Figure 1A). No such difference was seen between non-responders and responders in the pembrolizumab and ipilimumab/nivolumab combination groups (Figures 1B,C).

**Figure 2:**
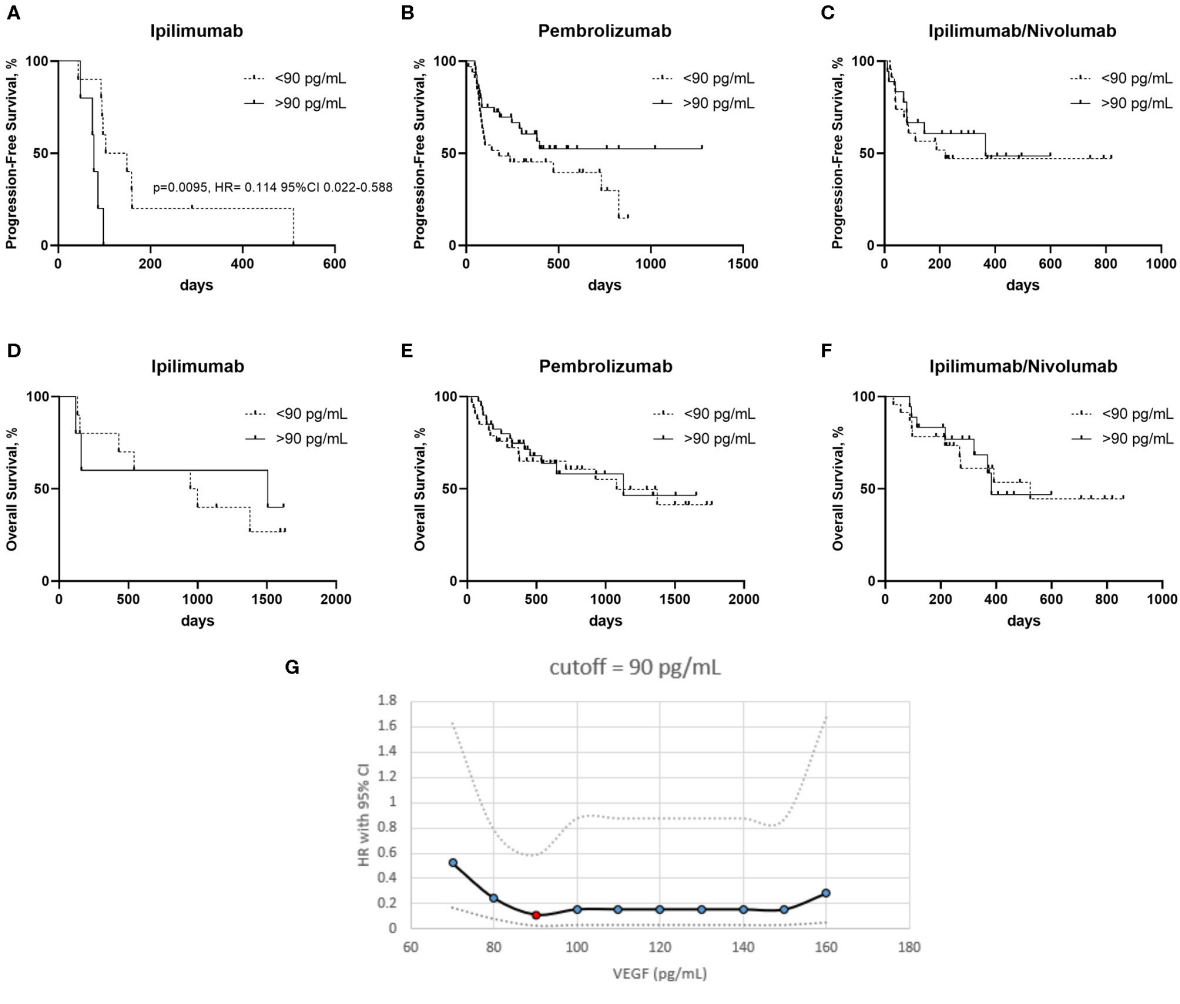
Kaplan–Meier curves of patients treated with ipilimumab **(A,D)** pembrolizumab **(B,E)** and ipilimumab/nivolumab **(C,F)**, with high and low serum VEGF levels on progression free survival (PFS) **(A–C)** and overall survival (OS) **(D–F)**. **(G)** Cut-off finder curves representing the hazard rations (HR) and 95% Confidence Intervals (CI) obtained a t multiple cut-off values from 70 to 160 pg/mL of serum VEGF.

Progression free survival (PFS) was worse in the ipilimumab treated patients with a serum VEGF level >90 pg/mL vs. those with a lower serum VEGF level (<90 pg/mL) (Figure 2A). No significant differences in PFS were observed in the pembrolizumab and ipilimumab/nivolumab cohorts based on serum VEGF levels (Figures 2B,C). No differences in overall survival were observed in any of the groups (Figures 2D–F). Cut-off finder curves representing multiple cut-off values for serum VEGF levels are demonstrated in Figure 2G.

## Discussion

Blood based biomarkers are becoming increasingly popular in immuno-oncology, being relatively non-invasive and allowing longitudinal sample collection enabling us to potentially track an evolving immune response (6). A number of routine blood based biomarkers including serum lactate dehydrogenase (LDH), neutrophil lymphocyte ratio, serum albumin and C reactive protein (CRP) have been evaluated for their prognostic significance in previous studies (7, 8, 13, 14).

Serum VEGF level has been previously shown to be a negative prognostic biomarker in ipilimumab treated patients (9). Here we evaluated the prognostic biomarker significance of pre-treatment serum VEGF levels in three different ICI treated cohorts including those treated with anti-CTLA4 monotherapy, the newer anti-PD1 agents as well as combination anti-PD1/anti-CTLA4 therapy. Our results indicate reduced likelihood of benefit from ipilimumab in the context of high serum VEGF levels as demonstrated by Yuan et al. (9) in their study.

Serum VEGF levels have also been previously evaluated as a predictive biomarker of response to identify patients more suitable for treatment with interleukin 2 (IL-2) in advanced melanoma (15). In this study serum VEGF and fibronectin were identified as independent predictors of response. In particular, high levels of serum VEGF correlated with reduced likelihood of clinical response and poor overall survival with IL-2.

To our knowledge, the role of serum VEGF as a predictive biomarker of response to anti-PD1 alone or in combination with ipilimumab has not been previously evaluated. We did not see any strong association between serum VEGF levels and response in advanced melanoma patients treated with pembrolizumab or combination ipilimumab and nivolumab. In these two cohorts, there was no difference in median serum VEGF levels amongst responders and non-responders and the PFS as well as OS were similar in the two sub-groups based on serum VEGF levels.

In the Checkmate-067 phase 3 study, the greatest benefit of combination ICI therapy was seen in the PD-L1 negative, BRAF mutant and elevated LDH subgroups (16). Serum VEGF levels should be further evaluated in a larger prospective study and potentially help us identify a proportion of advanced melanoma patients who might benefit from combination ICI therapy rather than monotherapy. This will be a very useful finding as dual therapy is associated with significant immune related adverse events, often leading to hospitalization (16).

Vascular endothelial growth factor modulates anti-tumour immune responses through poor antigen presentation due to inhibition of dendritic cell maturation, interfering with T-cell trafficking and creating an immune suppressed tumour microenvironment through MDSCs and T-reg stimulation (10, 11, 17). Combination anti-PD1 and anti-VEGF therapies are being currently evaluated in a number of clinical trials across a range of tumour types due to the anticipated synergy from the combination approach. Recently, there have been a few positive studies reported in non-small lung cancer (18) and endometrial cancer (19) evaluating this combination approach. Therefore, a biomarker evaluating the VEGF pathway might be particularly useful in future especially due to its potential to identify the subset of patients who might be better suited to dual therapy rather single agent ICI therapy.

There are a number of limitations of our study. The sample size is small for the ipilimumab treated group, however, our results confirm previous observations (9). There are some differences in the baseline demographics between the cohorts as this was not a randomized study. With the more common use of first-line combination ipilimumab and nivolumab or other clinical trials in the second-line setting, the number of patients being treated with ipilimumab monotherapy has significantly reduced in the last few years. There is no validated cut off value for defining high vs. low serum VEGF levels, and we based our survival analysis on the best cut-off for the ipilimumab group. Nevertheless, using CutoffFinder we did not find a VEGF concentration cut-off to distinguish survival in patients treated with anti-PD-1 agents. We did not evaluate post treatment serum VEGF levels for comparison with treatment outcomes. However, Yuan et al. (9) did not find a significant change in serum VEGF after induction completion (week 12).

In conclusion, our results suggest that serum VEGF level in advanced melanoma patients may indicate poor response to ipilimumab, however it does not impact response to anti-PD-1 agents alone or in combination with ipilimumab. Future research could focus on evaluating the biomarker significance of serum VEGF levels in prospective clinical trials of ICI therapy.

## Data Availability Statement

The datasets generated for this study are available on request to the corresponding author.

## Ethics Statement

The studies involving human participants were reviewed and approved by Edith Cowan University Ethics Committee. The patients/participants provided their written informed consent to participate in this study.

## Author Contributions

MK and EG: study concept. MK, AA, and EG: data collection, analysis, interpretation. All authors contributed to the article and approved the submitted version.

## Conflict of Interest

The authors declare that the research was conducted in the absence of any commercial or financial relationships that could be construed as a potential conflict of interest.
